# Supplementary material for: Supramolecular copolymerization driven by integrative self-sorting of hydrogen-bonded rosettes
Source: Nat Commun. 2020 Apr 1;11:1623. doi: 10.1038/s41467-020-15422-6 (PMC7113319; doi:10.1038/s41467-020-15422-6)
Supplement: Supplementary file 1 — Supplementary Information [file 41467_2020_15422_MOESM1_ESM.pdf]

Supplementary Information

Supramolecular copolymerization driven by integrative self-sorting of hydrogen-bonded rosettes

Aratsu et al.

This PDF file includes

Supplementary Methods

Supplementary Figures

Supplementary Tables

Supplementary References

Supplementary Methods

Transmission electron microscopy (TEM): TEM images were acquired on JEM-2100F (JEOL) at an acceleration voltage at 120 kV. Samples were prepared by spin-coating MCH solutions onto carbon-coated STEM Cu grid (SHR-C075, grade: super ultrahigh resolution carbon; mesh, 339; whole size, 75 μm) and dried under vacuum for 24 h. The TEM observations were conducted without staining.

SAXS analysis: To verify the correct assignment of the SAXS features, scattering patterns have been simulated using "SPONGE": a materials science-oriented scattering pattern calculator written in Python 3. SPONGE allows the simulation of a scattering pattern from any three-dimensional solid, and allows for the inclusion of realistic characteristics such as polydispersity and structural dynamics. The SPONGE computes the theoretical scattering pattern of such a structure while staying as close as possible to first-principles of scattering. The structure has to be provided as a closed surface described using the common STL (STereo Lithography) file format, which can be output by the majority of 3D drawing software tools. Core to the SPONGE is the use of the Debye equation for calculating the scattering from an aggregate of randomly distributed, infinitesimally small points[S1], using a procedure very similar to that by Hansen[S2]. This is a computationally intensive equation as it scales with the square of the number of points, but very closely represents the physical interference phenomena underpinning the scattering process, and produces reliable data even to large Q. Other software, such as the Debyer library[S3], employ several approximations to make the calculation much less expensive, but can produce artefacts at the extrema in Q. We therefore choose to stay with the computationally more expensive, but simpler and more fundamental implementation to minimize potential sources of error.

After loading a 3D surface, the SPONGE exploits the VTK library methods to place random points inside a rectangular parallelepiped enveloping the 3D shape, and subsequently checks which of these points lie inside the closed 3D surface of the object. This process continues until a sufficient number of points are found to lie within the 3D shape, points that lie outside the shape are discarded. The Euclidian distance between every possible point pair is computed using a fast SciPy implementation [Jones E, Oliphant E, Peterson P, et al. SciPy: Open Source Scientific Tools for Python, 2001-, http://www.scipy.org/ [Online; accessed 2019-02-26].], and the Debye formula for points is applied to that to get the scattered intensity as a function of the scattering vector Q. The scattered intensity is scaled proportional to the square of the volume. This process is repeated a number of times, i.e. a new set of points is picked, and the intensity from the new point set is calculated. The mean intensity from all repetitions is then presented, with the standard deviation used as an estimate for the uncertainty for each point.

A number-weighted size distribution can be taken into account. The SPONGE currently implements a Gaussian size distribution, by picking a random scaling factor from a Gaussian

distribution for each independent repetition. This would be similar to probing a multitude of objects of different size. The simulation is not scaled to absolute scattering cross-section at the moment. To study the effect of a dynamic motion, the scattering patterns of a number of intermediate conformations (frames) are computed and averaged afterwards.

To simulate the helicoid structures, the following parameters were used: the Q vector is logarithmically divided into 200 points between 0.05 and 5 nm^{-1}, simulations were performed with 3000 points inside each object. Unless otherwise stated, the size distribution has a width (sigma) of 5%. Each simulated dataset is the average of 100 independent repetitions, and with these parameters takes about 20 minutes on a MacBook air running on four simultaneous threads of a 2.2 GHz Intel Core i7 (Broadwell, 5650U), with 8 GB of 1600 MHz DDR3 RAM. No GPU acceleration could be exploited for this configuration. The "best-match" scattering pattern was simulated with 200 repetitions using 10000 points in each object.

Synthesis methods: Compound **2**, **2S** and **1S** was prepared according to Supplementary Figure 1, 2 and 3, respectively. Compound **1**, **3**, and **pnp** were prepared according to reported procedures[S4,S5].

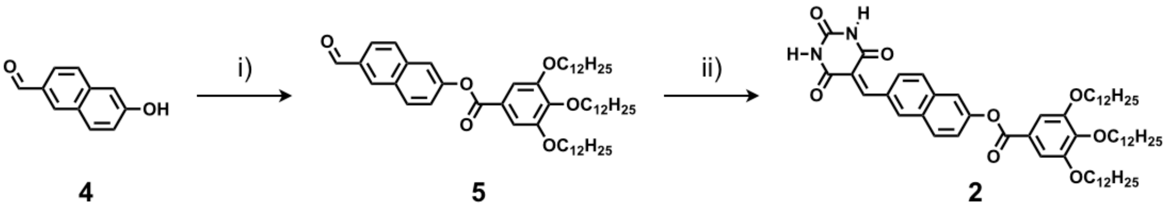

4 **5** **2**

Supplementary Figure 1. Synthetic scheme of compound **2**. i) 3,4,5-tri(*n*-dodecyloxy)benzoic acid, DCC, DMAP, CH_2Cl_2, r.t.; ii) barbituric acid, EtOH, reflux.

Synthesis of compound 5: Compound **4** (255 mg, 1.48 mmol), 3,4,5-tri(*n*-dodecyloxy)benzoic acid (1000 mg, 1.48 mmol) and *N,N*-dimethyl-4-aminopyridine (DMAP, 23.5 mg, 0.19 mmol) were dissolved in dry CH_2Cl_2 (10 mL). A solution of *N,N'*-dicyclohexylcarbodiimide (DCC, 455 mg, 2.21 mmol) in dry CH_2Cl_2 (5 mL) was added dropwise at 0 °C, and the mixture was stirred for overnight at room temperature under N_2 atmosphere. After the resulting white precipitates were filtered off, the filtrate was diluted with CH_2Cl_2 and washed with water and brine. The organic layer separated was dried over Na_2SO_4 and then evaporated to dryness under a reduced pressure. The resulting solid was purified by column chromatography over silica gel (eluent: $CHCl_3$) to give **5** as a white solid (1.32 g, 92% yield). ^{1}H NMR (500 MHz, $CDCl_3$, r.t.): δ 10.14 (1H, s, Ar-CH_aO), 8.34 (1H, s, Ar-H_b), 8.08 (1H, d, J = 8.8 Hz, Ar-H_c), 7.97 (1H, dd, J = 8.5, 1.5 Hz, Ar-H_d), 7.90 (1H, d, J = 8.8 Hz, Ar-H_e), 7.74 (1H, d, J = 2.2 Hz, Ar-H_f), 7.47–7.44 (3H, m, Ar-$H_{g,h}$), 4.11–4.05 (6H, m, Ar-OCH_i), 1.87–1.73 (6H, m, CH_j), 1.53–1.47 (6H, m, CH_k), 1.37–1.18 (48H, m, CH_l), 0.90–0.87 (9H, m, CH_m). ^{13}C NMR (125 MHz, $CDCl_3$, r.t.): δ 191.95, 164.94, 153.11, 151.35, 143.35, 137.06, 134.22, 134.10, 131.15, 130.69, 128.86, 123.67, 123.53, 122.84, 119.26, 108.70, 73.55, 69.22, 65.09, 31.92, 31.90, 30.35, 29.73, 29.68, 29.64, 29.62, 29.56, 29.38, 29.35, 29.27, 26.07, 22.67, 14.09. HRMS (APCI): *m/z* calcd for $C_{54}H_{85}O_6H$ 829.6341 [M+H]$^+$, found 829.6330.

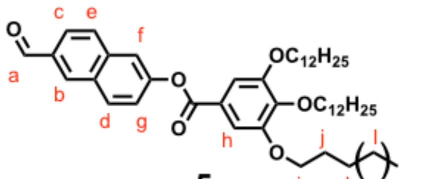

5

Synthesis of compound 2: A mixture of **5** (500 mg, 0.603 mmol) and barbituric acid (386 mg, 3.01 mmol) in EtOH (20 mL) was stirred for 12 h under reflux. The reaction mixture was cooled to room temperature and the resulting precipitates were collected by filtration and washed with hot ethanol repeatedly to give pure compound **2** as yellow solid (525 mg, 50%). ^{1}H NMR (500 MHz, $CDCl_3$, r.t.): δ 8.81 (1H, s, C=CH_a), 8.74 (1H, s, Ar-H_b), 8.34 (1H, s, NH_c), 8.27 (1H, d, J = 8.5, Hz, Ar-H_d), 8.15 (1H, s, NH_e), 8.03 (1H, d, J = 8.9 Hz, Ar-H_f), 7.88 (1H, d, J = 8.9 Hz, Ar-H_g), 7.72 (1H, d, J = 2.2 Hz, Ar-H_h), 7.45 (2H, s, Ar-H_i), 7.08 (1H, d, J = 2.2 Hz, Ar-H_j), 4.10–4.05 (6H, m, Ar- OCH_k), 1.87–1.75 (6H, m, CH_l), 1.51–1.47 (6H, m, CH_m), 1.37–1.26 (48H, m, CH_n), 0.90–0.86 (9H, m, CH_o). ^{13}C NMR (125 MHz, $CDCl_3$, r.t.): δ 164.88, 163.76, 161.30, 159.60, 152.95, 151.67, 150.15, 143.17, 138.07, 136.32, 131.71, 130.41, 129.55, 127.59, 123.40, 122.49, 118.76, 115.96, 108.53, 73.57, 69.23, 31.96, 31.94, 30.42, 29.80, 29.74, 29.69, 29.49, 29.43, 29.39, 26.15, 26.12, 22.70, 14.13. HRMS (APCI): m/z calcd for $C_{58}H_{86}N_2O_8H$ 939.6457 $[M+H]^+$, found 939.6446.

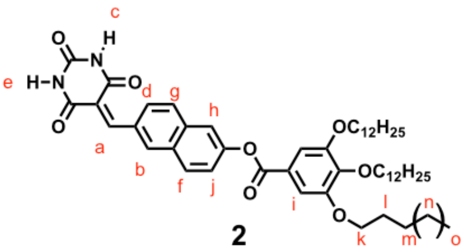

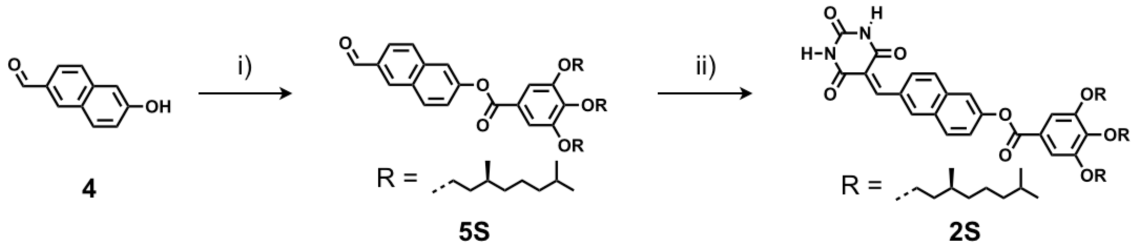

Supplementary Figure 2. Synthetic scheme of compound **2S**. i) 3,4,5-tris(((*S*)-3,7-dimethyloctyl)oxy)benzoic acid, DCC, DMAP, CH_2Cl_2, r.t.; ii) barbituric acid, EtOH, reflux.

Synthesis of compound 5S: Compound **4** (99 mg, 0.58 mmol), 3,4,5-tris(((*S*)-3,7-dimethyloctyl)oxy)benzoic acid (334 mg, 0.565 mmol) and *N,N*-dimethyl-4-aminopyridine (DMAP, 8 mg) were dissolved in dry CH_2Cl_2 (10 mL). A solution of *N,N'*-dicyclohexylcarbodiimide (DCC, 118 mg) in dry CH_2Cl_2 (5 mL) was added dropwise at 0 °C, and the mixture was stirred for overnight at room temperature under N_2 atmosphere. After the resulting white precipitates were filtered off, the filtrate was diluted with CH_2Cl_2 and washed with water and brine. The organic layer separated was dried over Na_2SO_4 and then evaporated to dryness under a reduced pressure. The resulting solid was purified by column chromatography over silica gel (eluent: $CHCl_3$) to give **5S** as a viscous transparent liquid (92 mg, 22% yield). ^{1}H NMR (300 MHz, $CDCl_3$, r.t.): δ 10.18(1H, s), 8.39 (1H, s), 8.10–8.07

(1H, d, J = 9.0 Hz), 8.02–7.99 (1H, d, J = 9.0 Hz), 7.99–7.92 (1H, d, J = 9.0 Hz), 7.76–7.75 (1H, m), 7.49–7.48 (1H, m), 7.46 (2H, s), 4.12–4.08 (6H, m), 1.94–1.83 (3H, m), 1.71–1.48 (9H, m), 1.37–1.15 (18H, m), 0.97–0.93 (9H, m), 0.89–0.85 (18H, m).

Synthesis of compound 2S: A mixture of **5S** (162 mg, 0.217 mmol) and barbituric acid (228 mg, 1.78 mmol) in EtOH (30 mL) was stirred for 3 h under reflux. The reaction mixture was cooled to room temperature, and the resulting precipitates were collected by filtration and washed with hot ethanol repeatedly to give pure compound **2S** as yellow solid (77 mg, 40%). ^{1}H NMR (500 MHz, $CDCl_3$, r.t.): δ 8.80 (1H, s), 8.75 (1H, s), 8.31–8.28 (1H, d, J = 8.7 Hz), 8.20 (1H, s), 8.06 (1H, s), 8.03 (2H, s), 7.90–7.88 (1H, d, J = 8.7 Hz), 7.73 (s, 1H), 7.46 (2H, s), 7.44–7.43 (1H, d, J = 2.3 Hz), 4.12–4.08 (6H, m), 1.92–1.83 (3H, m), 1.72–1.65 (6H, m), 1.55–1.48 (3H, m), 1.34–1.31 (6H, m), 1.27–1.22 (6H, m), 1.19–1.15 (6H, m), 0.97–0.93 (9H, m), 0.89–0.85 (18H, m). ^{13}C NMR (125 MHz, $CDCl_3$, 60 °C): δ 164.87, 163.20, 160.85, 159.86, 153.21, 151.97, 149.28, 143.98, 137.62, 136.56, 131.64, 130.63, 130.23, 129.75, 127.71, 123.63, 122.61, 118.83, 116.25, 109.33, 109.26, 68.05, 67.95, 39.50, 39.40, 37.63, 37.51, 36.71, 36.57, 30.22, 30.06, 29.89, 28.04, 24.77, 22.90, 22.68, 22.60, 19.68. HRMS (APCI): m/z calcd for $C_{52}H_{74}N_2O_8H$ 939.6457 $[M+H]^+$, found 939.6446.

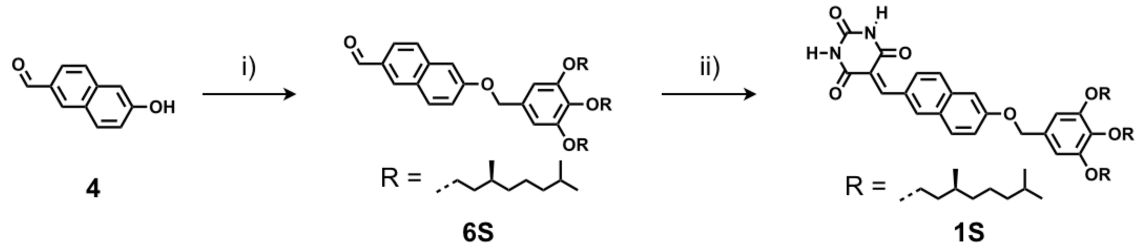

Supplementary Figure 3. Synthetic scheme of compound **1S**. i) 3,4,5-tris(((*S*)-3,7-dimethyloctyl)oxy)benzoic acid, K_2CO_3, DMF, 65 °C.; ii) barbituric acid, EtOH, reflux.

Synthesis of compound 6S: Compound **4** (74 mg, 0.134 mmol) was dissolved in a suspension of K_2CO_3 (940 mg, 6.8 mmol) in DMF (5 mL) at 65 °C, and the mixture was stirred until the solution turned green. 3,4,5-Tris(((*S*)-3,7-dimethyloctyl)oxy)benzoic acid (23 mg, 0.134 mmol) dissolved in 5 mL of DMF was added dropwise and the mixture was stirred for 4 h at 65 °C. The reaction mixture was poured into ice-water and the resulting precipitates

were collected by filtration to afford **3** (43 mg, 55% yield). ^{1}H NMR (300 MHz, $CDCl_3$): δ 10.1 (s, 1H), 8.27 (s,1H), 8.02–7.92 (m, 2H), 7.82–7.79 (m, 1H), 7.35–7.32 (m, 1H), 7.31–7.28 (m, 1H), 6.69 (s, 2H), 5.11 (s, 2H), 4.06–3.95 (m, 6H), 1.89–1.48 (m, 12H), 1.35–1.14 (m, 18H), 0.94-0.85 (m, 27H).

Synthesis of compound 1S: A mixture of **6S** (26 mg, 0.035 mmol) and barbituric acid (45.5 mg, 0.035 mmol) in EtOH (10ml) was refluxed for 4 h under N_2 atmosphere. The reaction mixture was cooled to r.t. and the resulting precipitates were collected by filtration and washed with hot ethanol repeatedly to give pure compound **1S** as yellow solid (6 mg, 21%). ^{1}H NMR (300 MHz, $CDCl_3$): δ 7.96 (s, 1H), 7.90–7.83 (m, 4H), 7.72–7.69 (m, 2H), 7.53–7.49 (m, 2H), 6.88 (s, 2H) 4.89–4.87 (m, 2H), 4.10–3.99 (m, 6H), 1.89–1.74 (m, 6H), 1.58-1.48 (m, 6H), 1.32-1.26 (m, 36H), 0.90–0.85 (m, 9H).

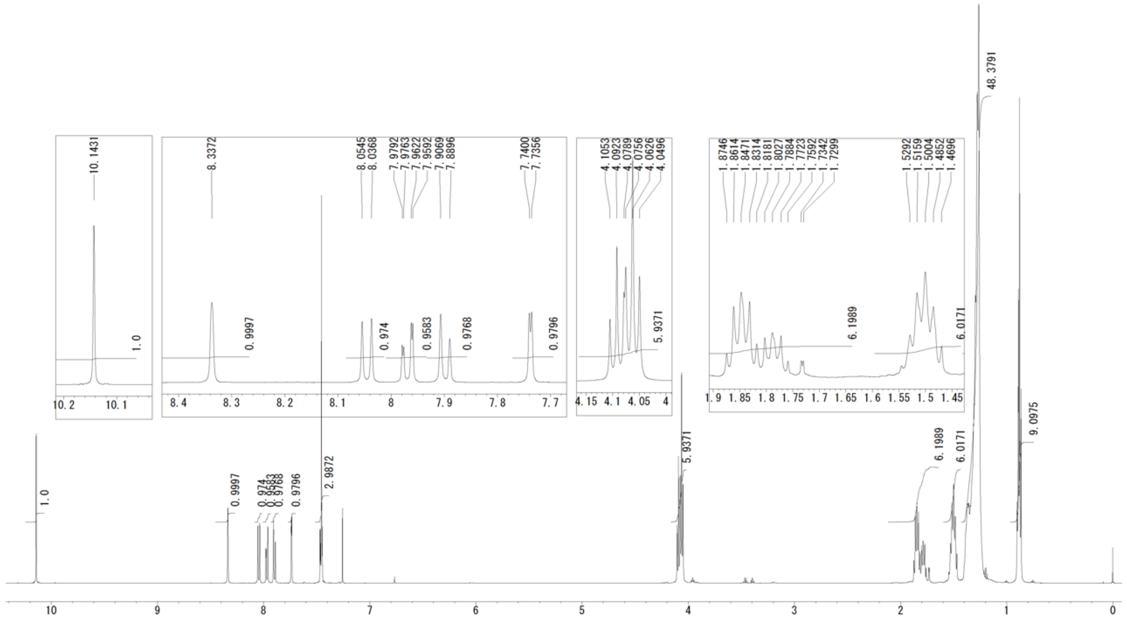

Supplementary Figure 4. ^{1}H NMR spectrum of **5** in $CDCl_3$.

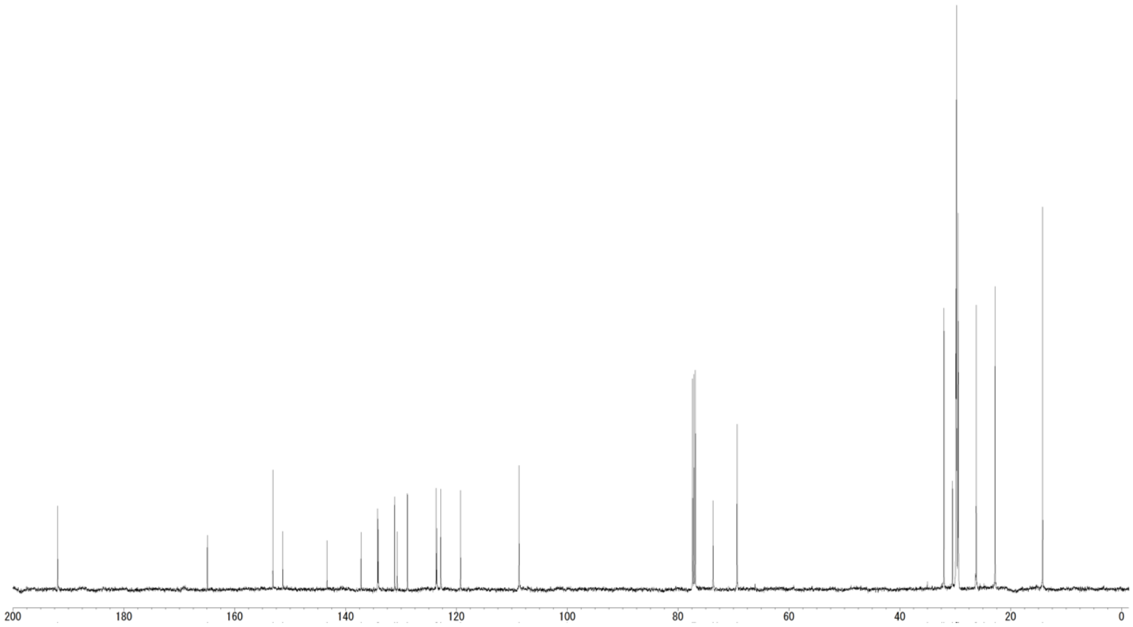

Supplementary Figure 5. ^{13}C NMR spectrum of **5** in $CDCl_3$.

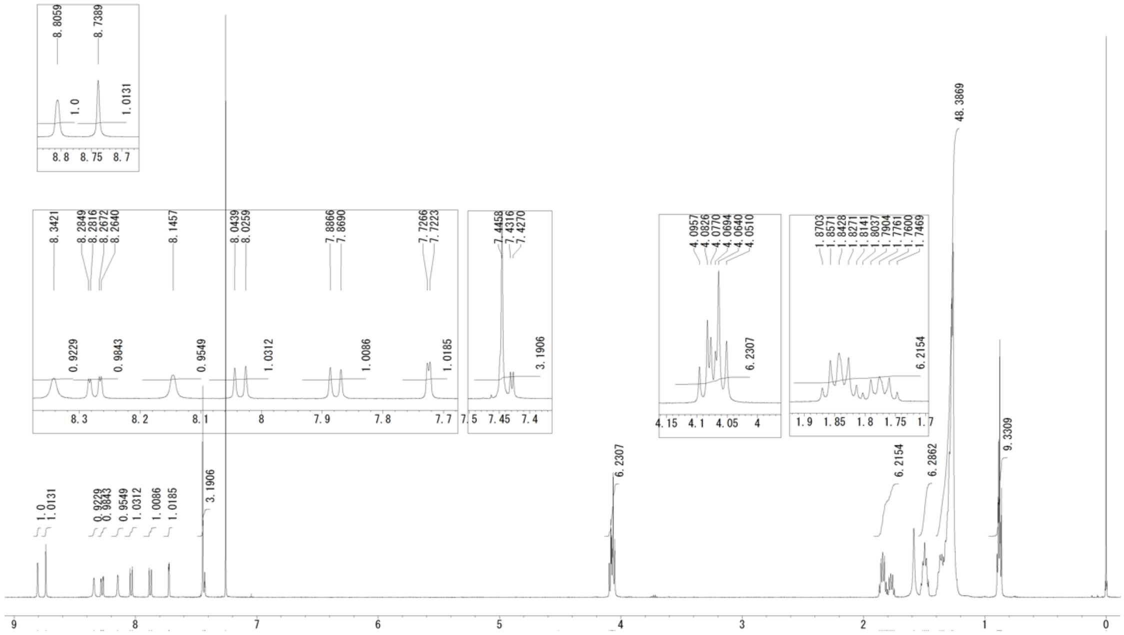

Supplementary Figure 6. ^{1}H NMR spectrum of **2** in $CDCl_3$.

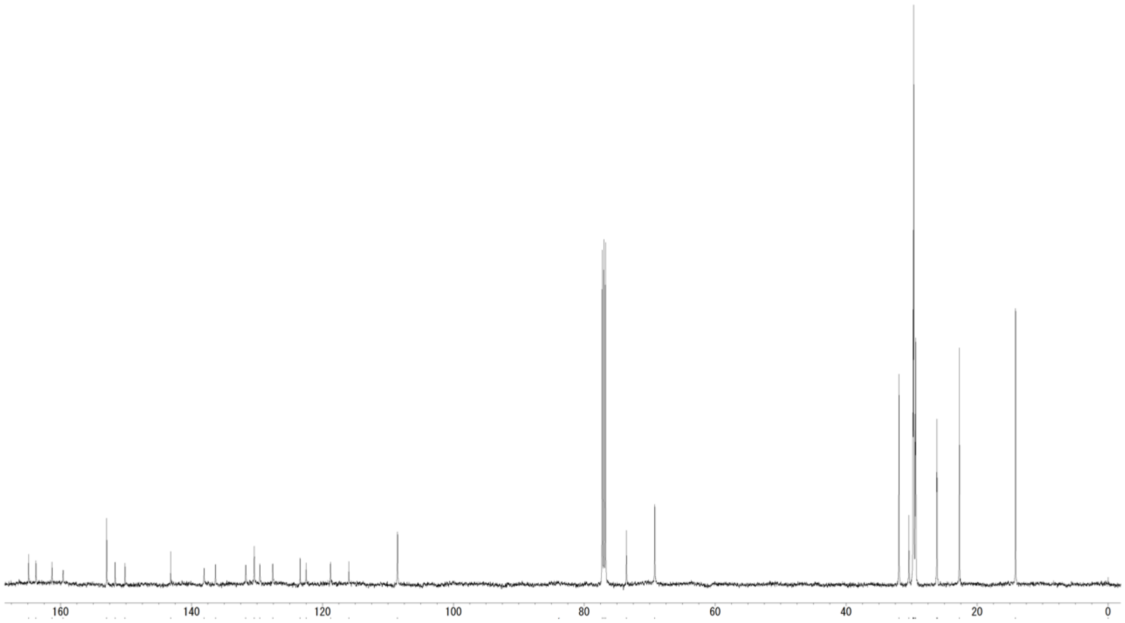

Supplementary Figure 7. ^{13}C NMR spectrum of **2** in $CDCl_3$.

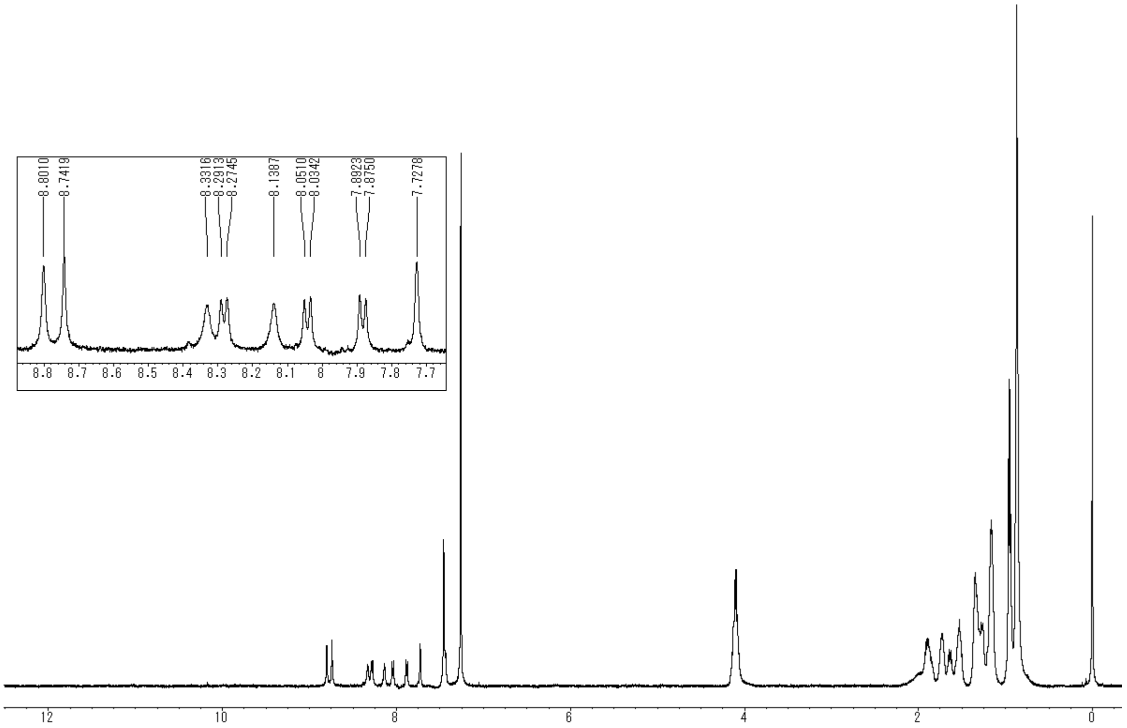

Supplementary Figure 8. ^{1}H NMR spectrum of **2S** in $CDCl_3$.

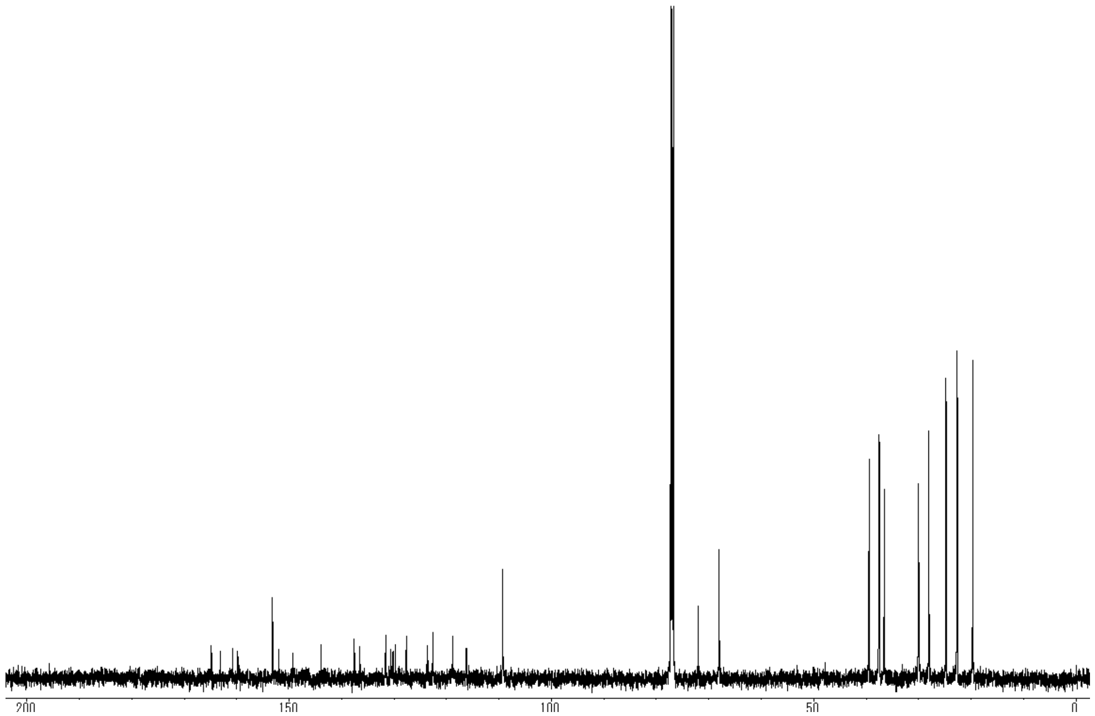

Supplementary Figure 9. ^{13}C NMR spectrum of **2S** in $CDCl_3$.

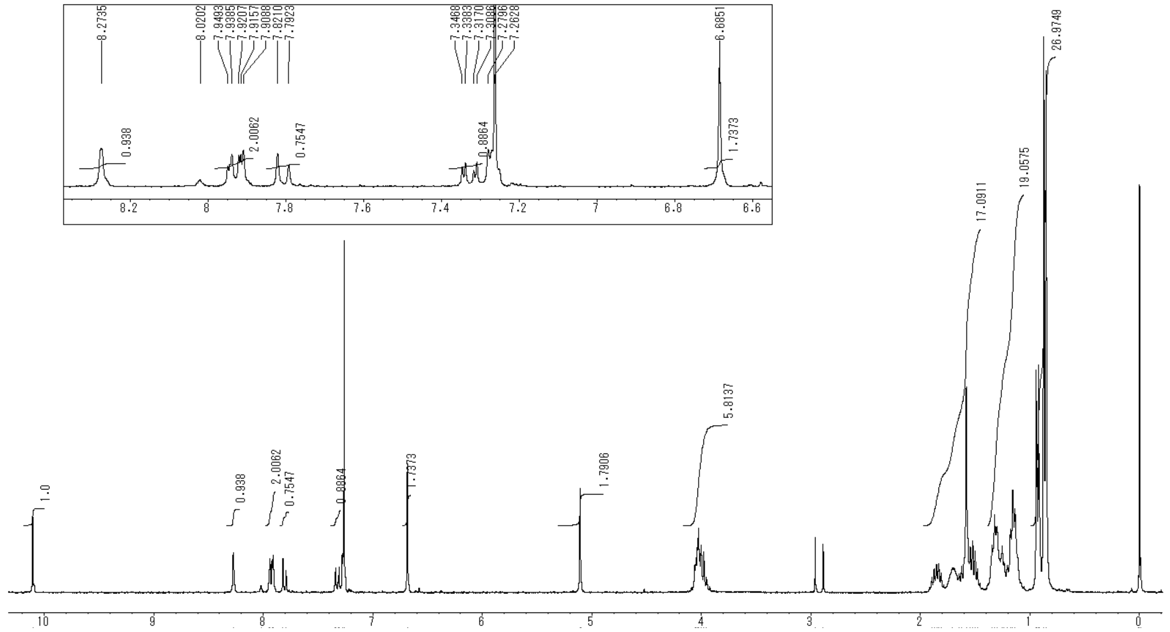

Supplementary Figure 10. ^{1}H NMR spectrum of **6S** in $CDCl_3$.

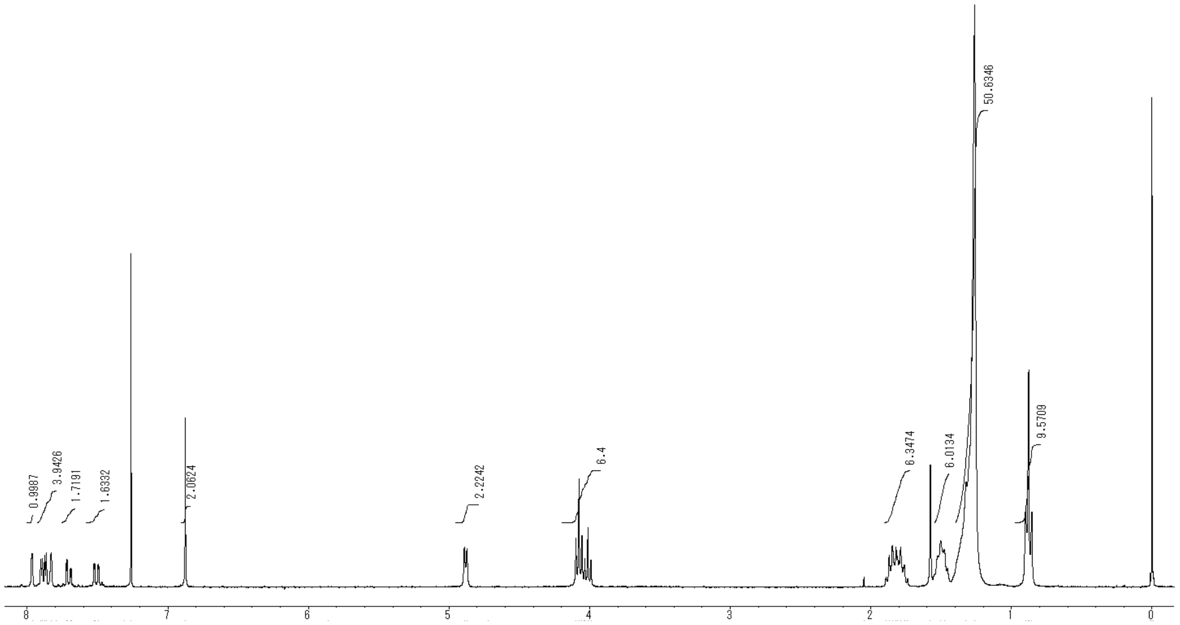

Supplementary Figure 11. ^{1}H NMR spectrum of **1S** in $CDCl_3$.

Supplementary Figures

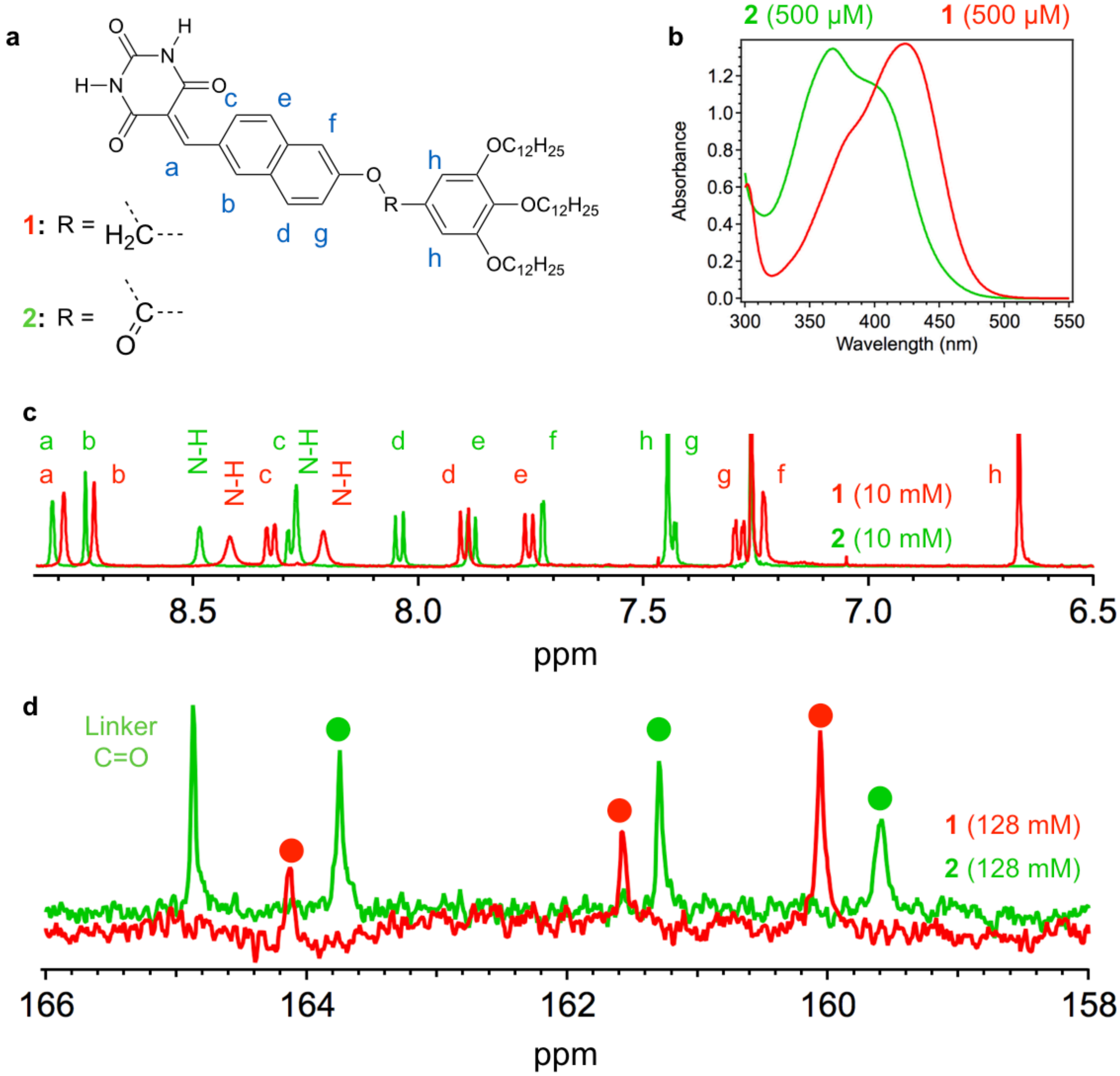

Supplementary Figure 12. Comparison of UV–vis absorption and NMR spectra of monomeric 1 and 2 in CH(D)Cl3. a, Proton-labeled molecular structures of **1** and **2**. **b**, UV–vis absorption spectra of monomeric **1** (red) and **2** (green) in $CHCl_3$ at 20 °C; c_t = 500 μM. **c**,**d**, ^{1}H (**c**) and ^{13}C (**d**) NMR spectra of **1** (red) and **2** (green) in $CDCl_3$ at 20 °C; c_t = 10 mM for ^{1}H NMR spectra, and 128 mM for ^{13}C NMR spectra.

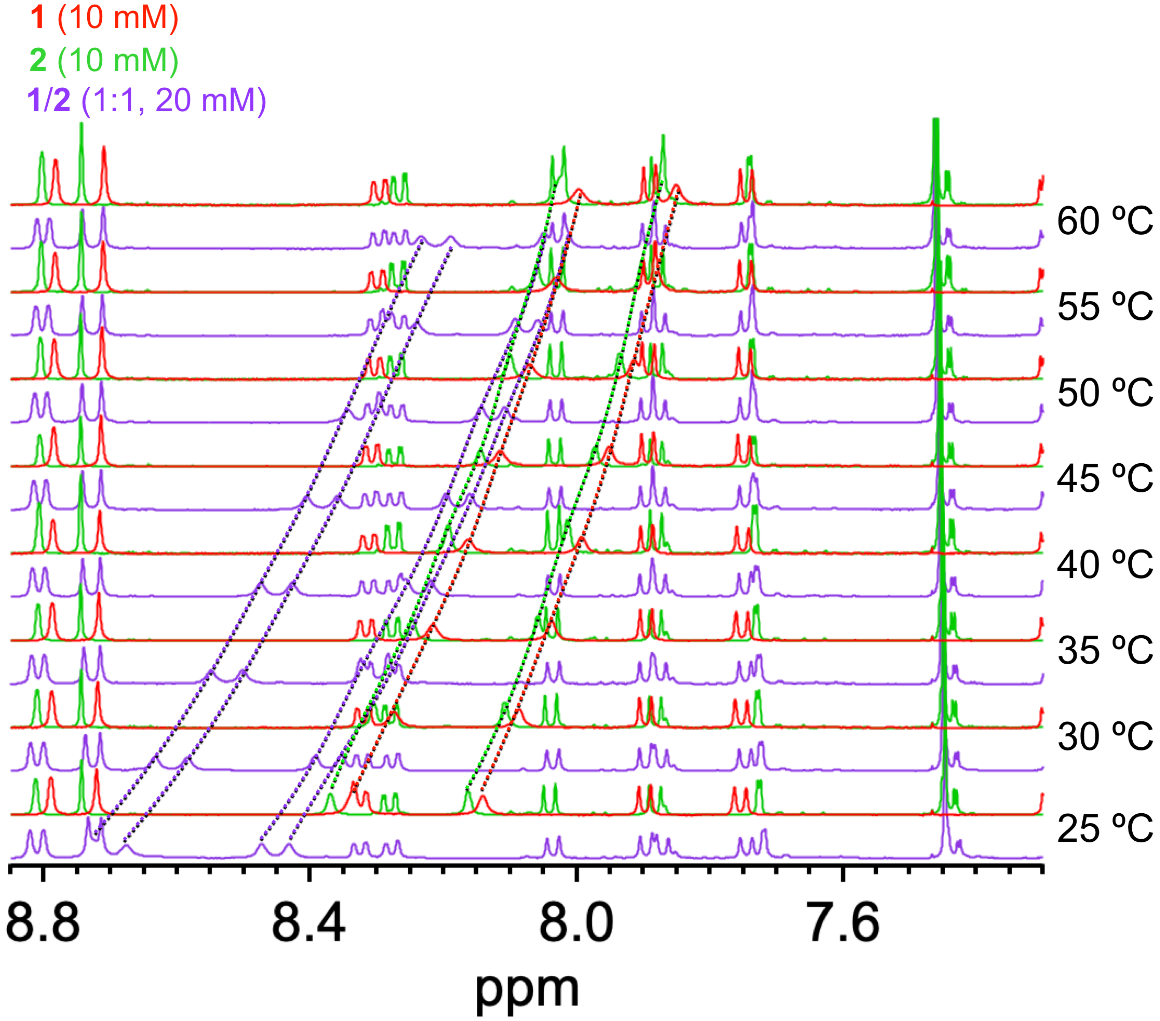

Supplementary Figure 13. Temperature-dependent ^{1}H NMR spectra in $CDCl_3$. Temperature-dependent ^{1}H NMR spectra of **1** (red, c_t = 10 mM), **2** (green, c_t = 10mM) and **1**/**2** (1:1, purple, c_t = 20 mM) in $CDCl_3$ upon cooling from 60 °C to 25 °C. Dashed lines show NH peak shifts.

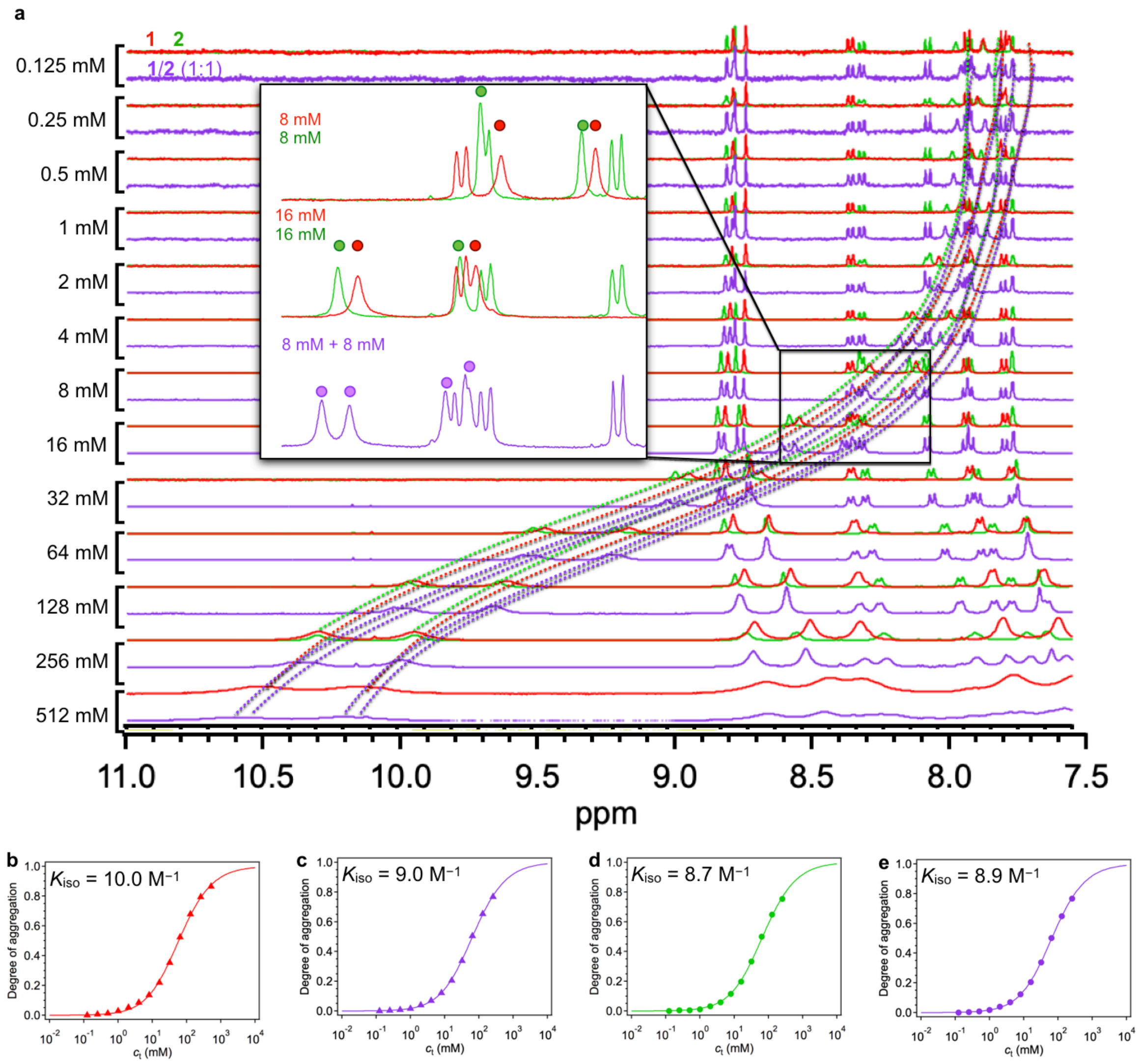

Supplementary Figure 14. Concentration-dependent ^{1}H NMR spectra. a, Concentration-dependent ^{1}H NMR spectra of **1** (red), **2** (green) and **1/2** (1:1, purple) in $CDCl_3$ at 20 °C. Dashed lines trace the shift of the two NH proton peaks of barbituric acid unit. These peaks shifted to down-field upon increasing the concentration. The shift was not saturation even 512 mM, indicative of a low association constant. Inset in (**a**) shows the comparison of NH proton peaks of **1/2** (c_t = 16 mM) with those of pure **1** and **2** at c_t = 8 and 16 mM. Because the peak positions of **1/2** (c_t = 16 mM) are almost the same with those of **1** and **2** at c_t = 16 mM, **1** and **2** in the mixture do not narcissistically self-sort. **b**–**e**, Plots of the degree of aggregation calculated from the chemical shifts of NH protons as a function of the concentration. The sigmoidal curves can be fitted with an isodesmic model to give isodesmic association constants (K_{iso}) of (**b**) 10.0 M^{-1} for pure **1**, (**c**) 9.0 M^{-1} for **1** in **1/2** (1:1), (**d**) 8.7 M^{-1} for pure **2** and (**e**) 8.9 M^{-1} for **2** in **1/2** (1:1)[S6].

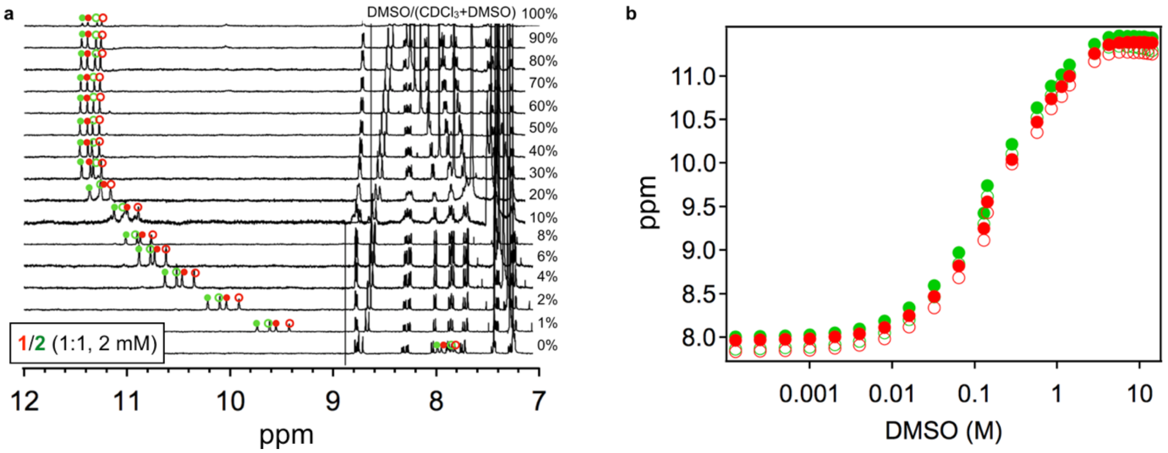

Supplementary Figure 15. Titration study. a, ^{1}H NMR spectra of **1/2** (1:1, c_t = 2 mM) in $CDCl_3$-DMSO-d_6 mixtures with various compositions. NH proton peaks shift to low-field upon increasing DMSO-d_6 content from 0% (bottom) to 100% (top) due to hydrogen-bonding with DMSO-d_6. The red and green circles correspond to the NH proton peaks of **1** and **2**, respectively. **b**, Transition of the chemical shift of NH proton peaks of **1** (red) and **2** (green) in **1/2** as a function of concentration of DMSO-d_6. Almost the same transition behavior of **1** and **2** demonstrates the identical hydrogen-bonding capability of their NH protons.

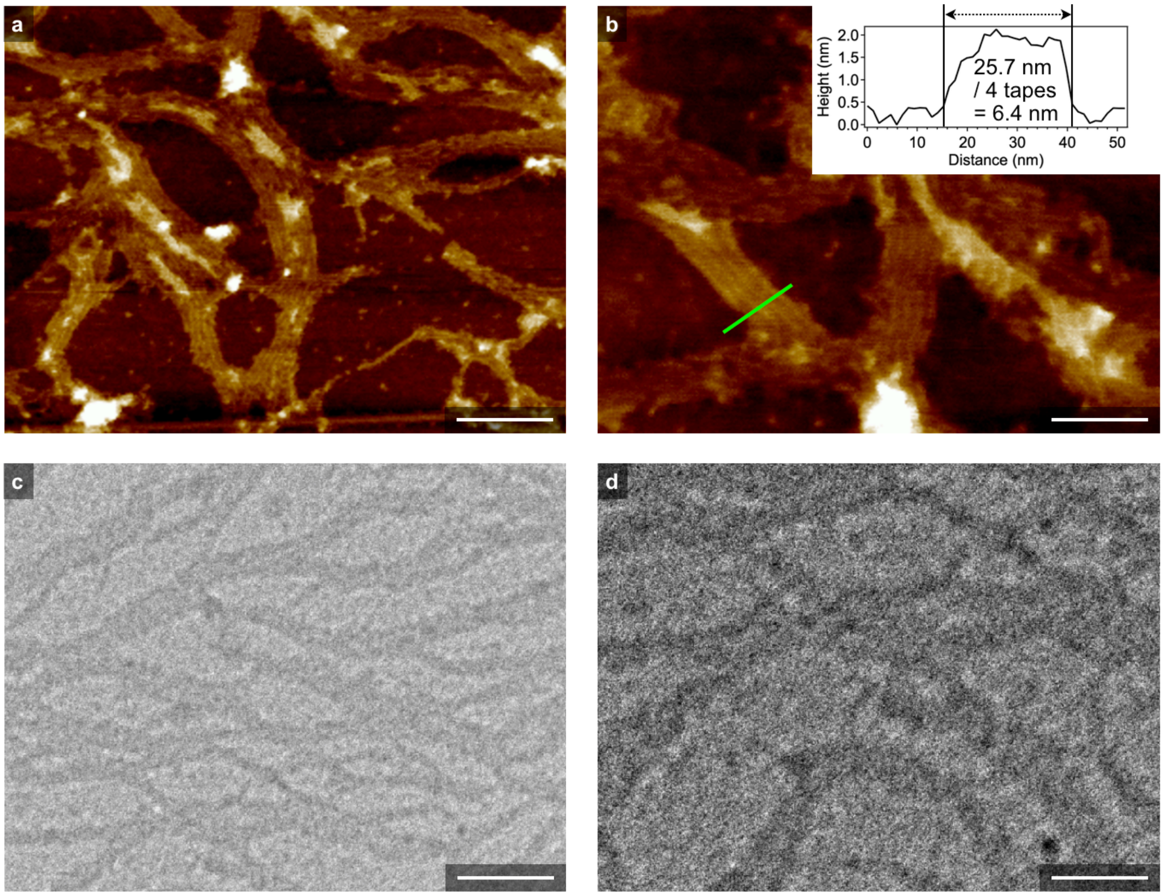

Supplementary Figure 16. AFM and TEM images of 2. AFM (**a**,**b**) and TEM (**c**,**d**) images of linear fibril of **2** (c_t = 250 μM). Inset in (**b**) is cross-sectional analysis along the green line. Scale bars, 100 nm (**a**,**c**) and 50 nm (**b**,**d**).

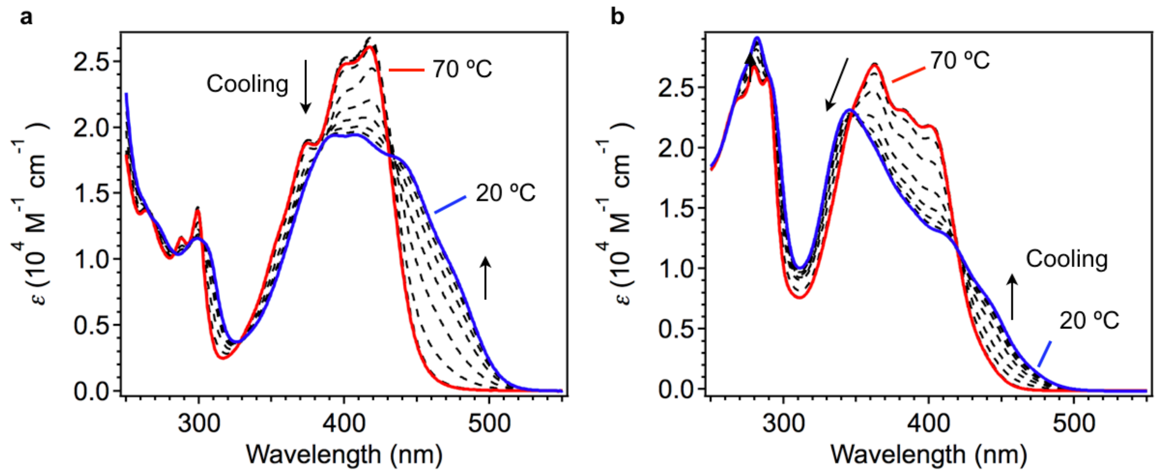

Supplementary Figure 17. Temperature-dependent UV–vis absorption study of 1 and 2. Temperature-dependent UV–vis absorption spectra of **1** (**a**) and **2** (**b**) at 50 μM upon cooling from 70 °C (red) to 20 °C (blue). Allows indicate spectral change upon cooling.

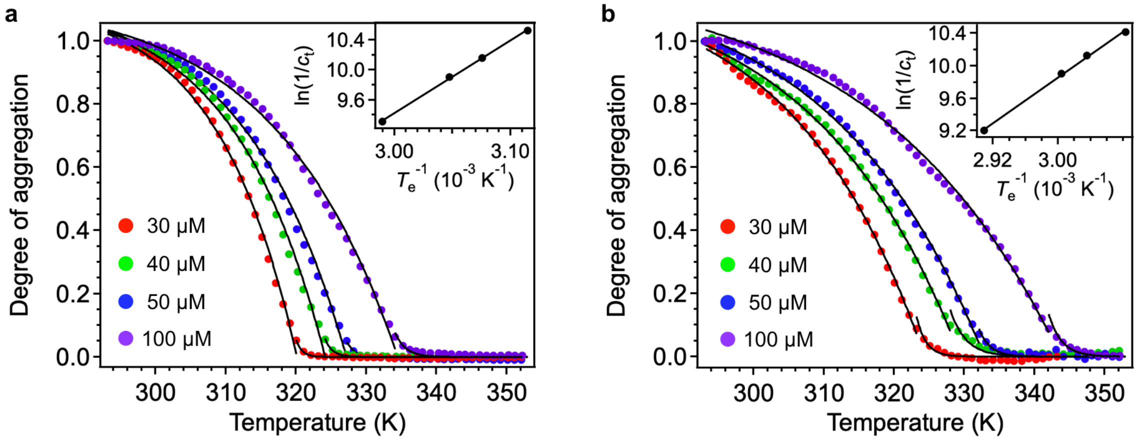

Supplementary Figure 18. Supramolecular polymerization processes of 1 and 2. Plots of the degree of aggregation of **1** (**a**) and **2** (**b**) in MCH at different concentrations (c_t = 30 μM, 40 μM, 50 μM, and 100 μM) calculated from the absorption change at λ = 470 nm as a function of the temperature during the cooling process (cooling rate: 1 K min^{-1}). The black solid lines were obtained by fitting the cooling curves to the cooperative model proposed by Meijer and co-workers[S7,S8]. Insets in (**a**,**b**) show van't Hoff plots using the natural logarithm of the reciprocal c_t and the reciprocal critical temperature (T_e).

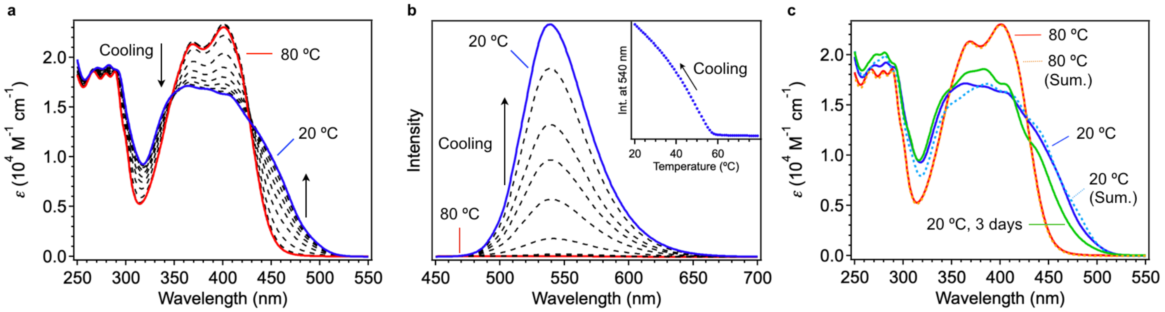

Supplementary Figure 19. Temperature-dependent UV–vis absorption and fluorescence spectra of 1/2. a,**b**, Temperature-dependent UV–vis absorption (**a**) and fluorescence emission spectra (**b**) of **1/2** (1:1, c_t = 100 μM) upon cooling from 80 °C (red) to 20 °C (blue) in MCH at a rate of 1 °C min^{-1}. Allows show change upon cooling. Inset in (**b**) shows a plot of temperature-dependent fluorescence emission change at 540 nm as a function of temperature. **c**, Comparison of the experimentally obtained absorption spectra of **1/2** (1:1, c_t = 100 μM) at 20 °C and 80 °C (solid lines) with those obtained by the summation of the individual spectra (dashed lines).

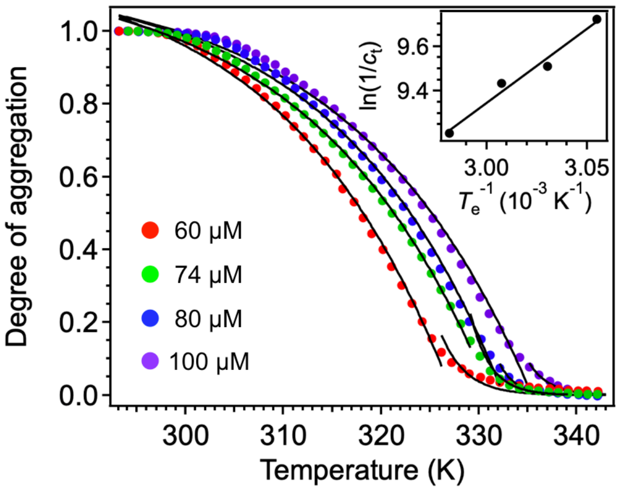

Supplementary Figure 20. Supramolecular polymerization process of 1/2. Plots of the degree of aggregation of **1/2** (1:1) in MCH at different concentrations (c_t = 60 μM, 74 μM, 80 μM, and 100 μM) as a function of temperature during the cooling process (cooling rate = 1 K min^{-1}). The black solid lines were obtained by fitting the cooling curves to the cooperative model proposed by Meijer and co-workers[S7,S8]. Inset shows a van't Hoff plot using the natural logarithm of the reciprocal c_t and the reciprocal critical temperature (T_e).

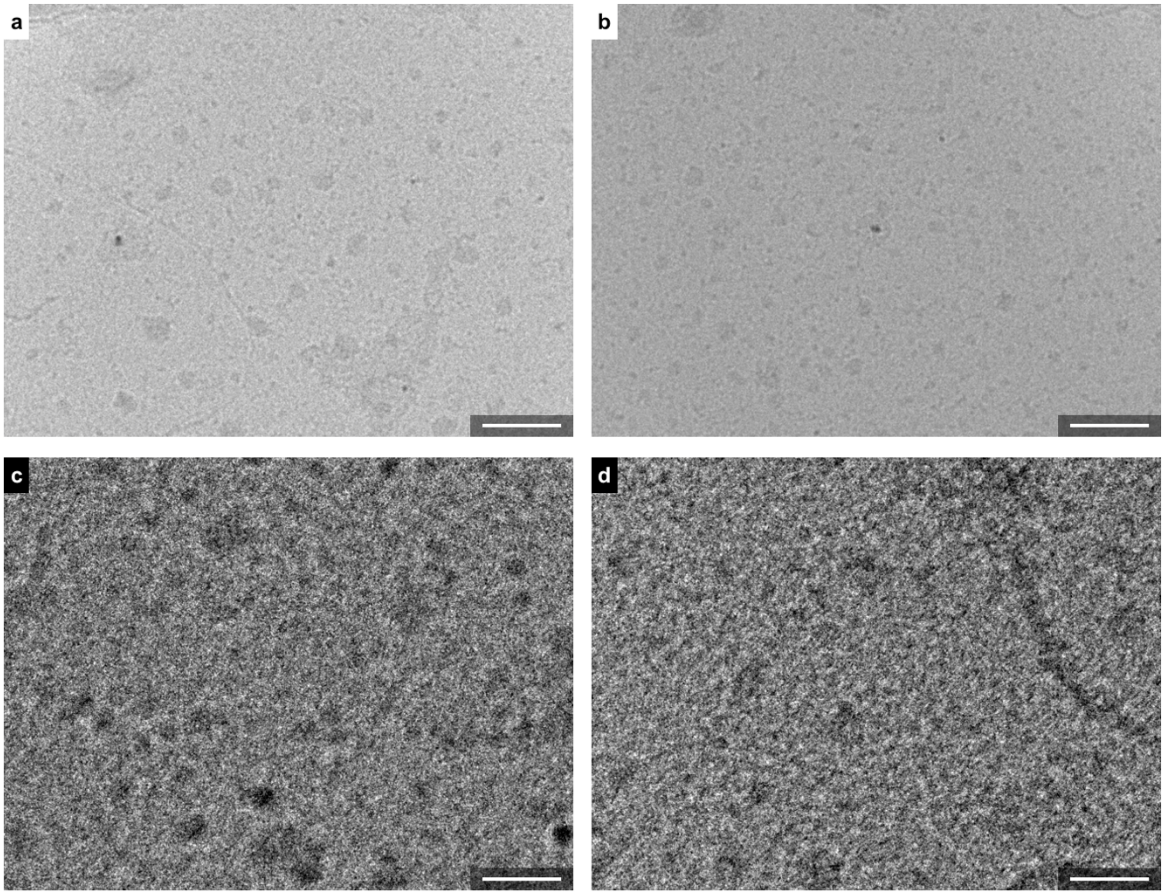

Supplementary Figure 21. TEM images of amorphous coaggregate. TEM images of amorphous coaggregates of **1/2** (1:1, c_t = 500 μM). The sample was prepared by spin-coating the as-cooled solution onto a carbon-coated copper grid. Scale bars, 200 nm (**a**,**b**) and 50 nm (**c**,**d**).

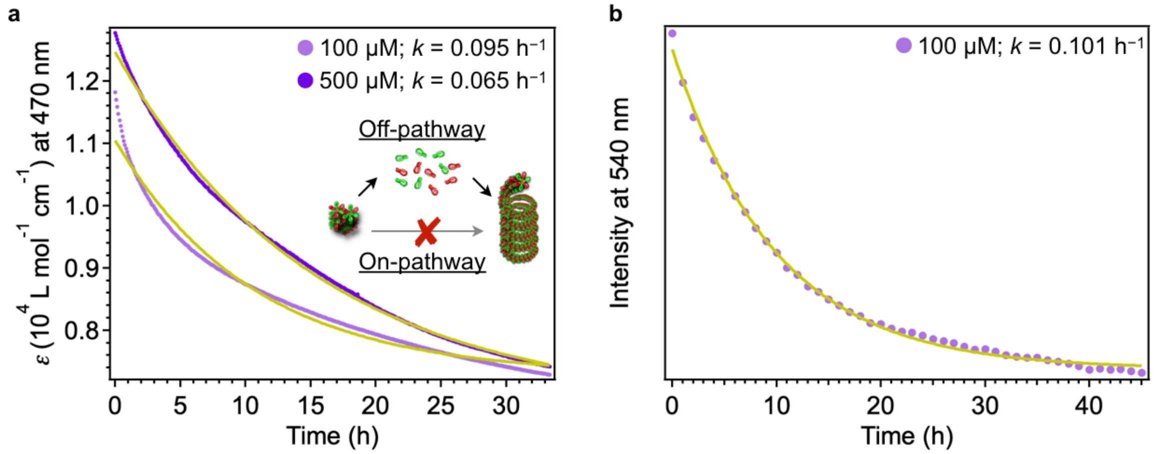

Supplementary Figure 22. Concentration-dependence of transformation kinetics of 1/2. Time-dependent change of the molar extinction coefficient (ε) at 470 nm (**a**) and the emission intensity at 540 nm (**b**) of **1/2** (1:1, c_t = 100 or 500 μM) in MCH at 20 °C. Yellow solid curves are the best fitting curves by a simple mono-exponential equation[S9].

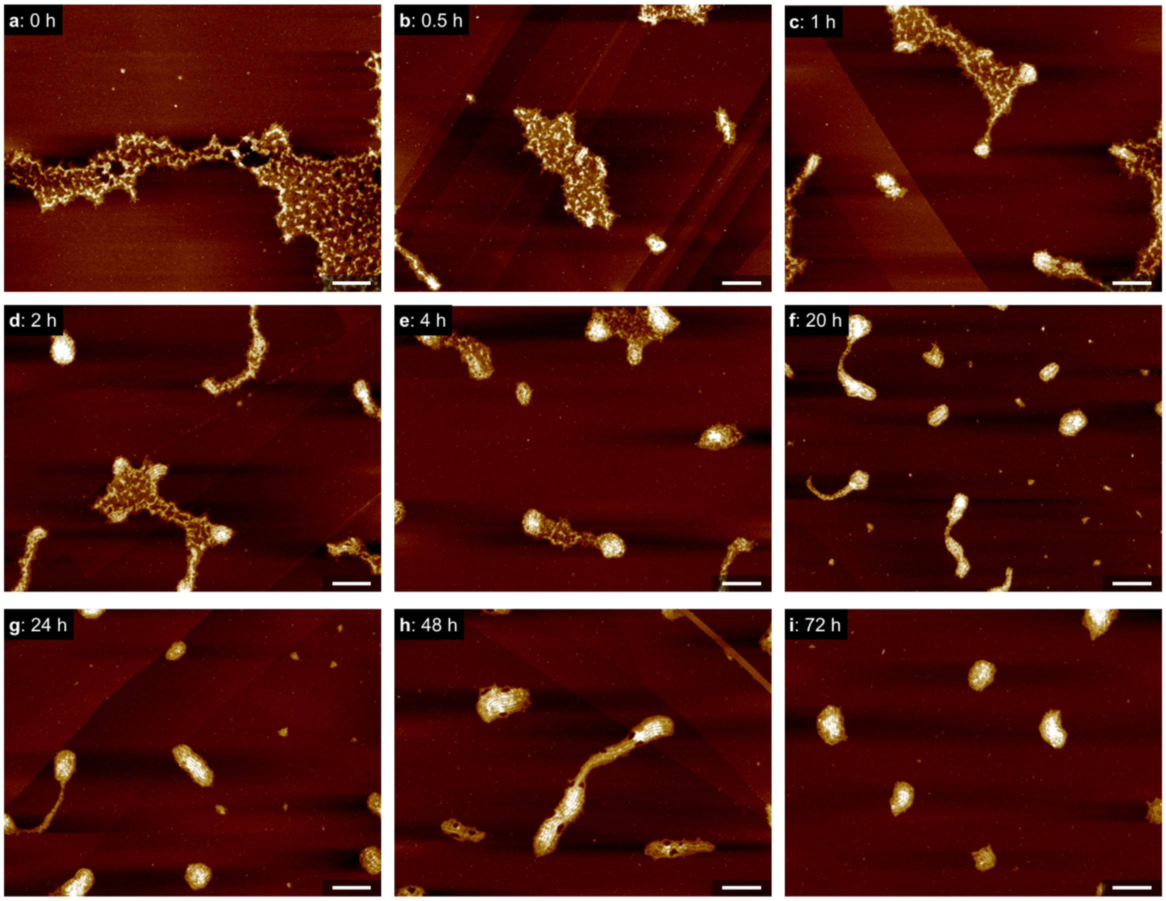

Supplementary Figure 23. Time-dependent AFM images showing transformation of 1/2 nanostructures. Time-dependent AFM images of **1/2** (1:1, c_t = 100 μM). The samples were prepared by spin-coating the solution onto HOPG after an equilibration time of 0 h (**a**), 0.5 h (**b**), 1 h (**c**), 2 h (**d**), 4 h (**e**), 20 h (**f**), 24 h (**g**), 48 h (**h**), and 72 h (**i**) at 20 °C. Scale bars, 300 nm.

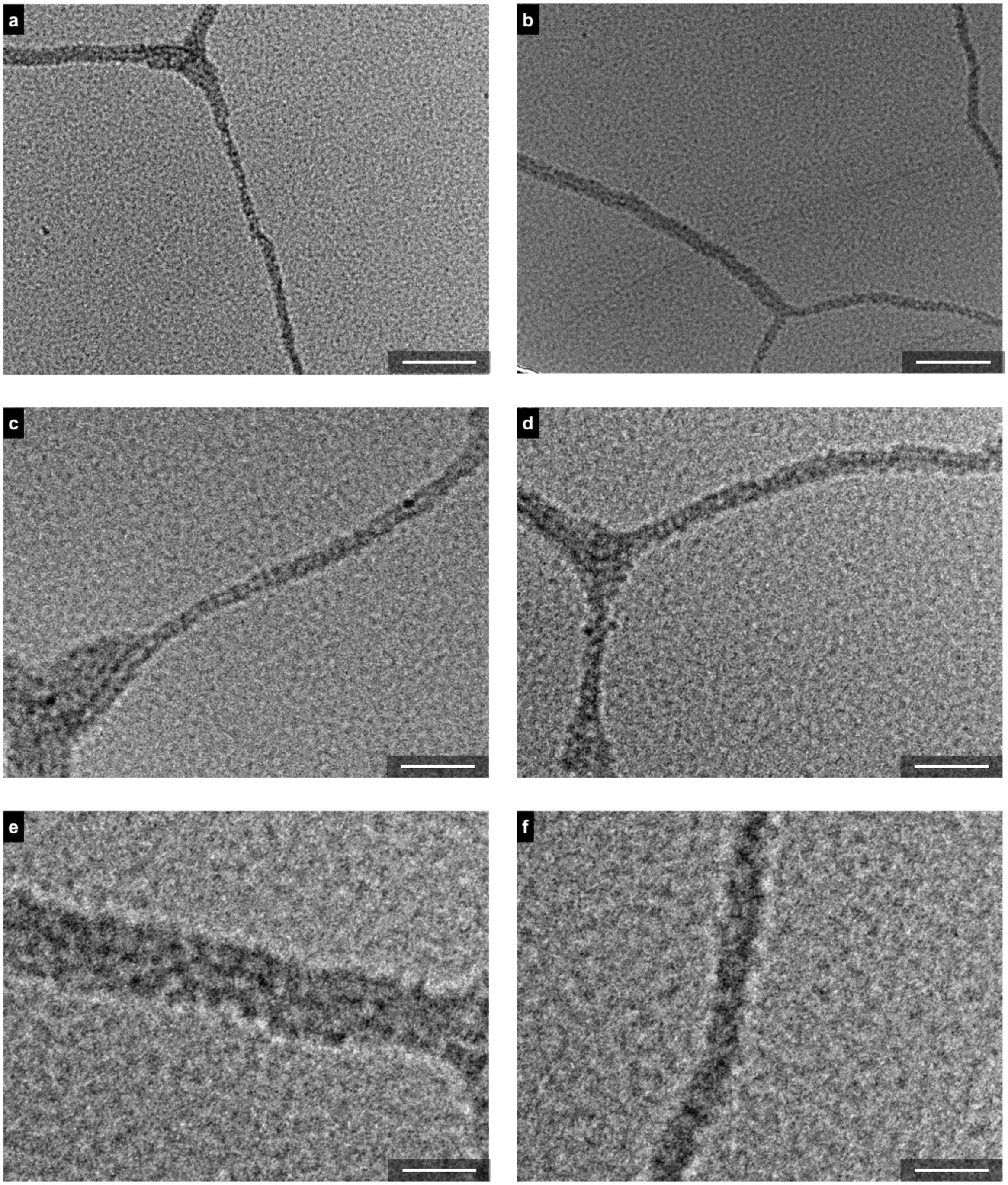

Supplementary Figure 24. TEM images of helicoids. TEM images of helicoidal structures of **1/2** (1:1, c_t = 500 µM). The samples were prepared by spin-coating the solution after an equilibration time of 168 h at 20 °C onto a carbon-coated copper grid. Scale bars, 200 nm (**a**,**b**), 100 nm (**c**,**d**), and 50 nm (**e**,**f**).

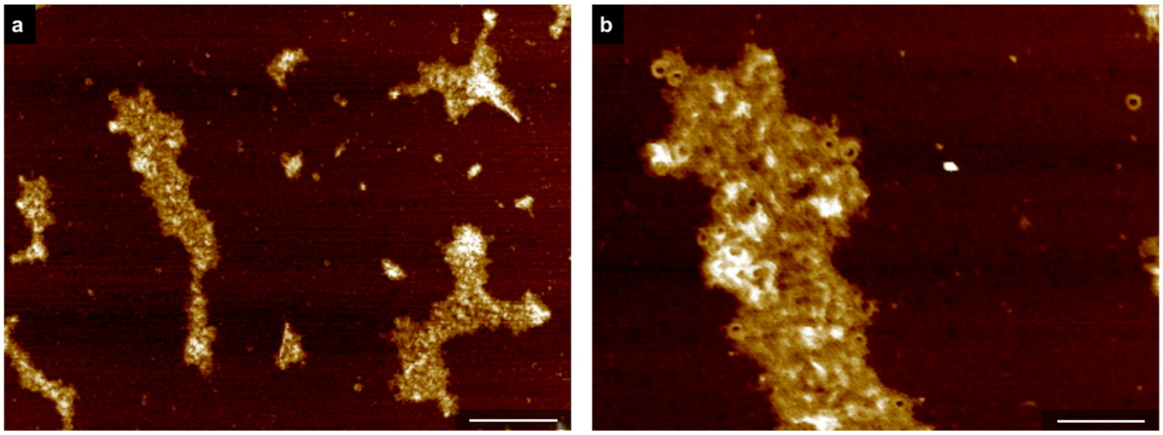

Supplementary Figure 25. AFM images of tertiary mixture. AFM images of **1/2/3** (1:1:1, c_t = 150 μM) spin-coated from the solution immediately after cooling. Scale bars, 300 nm (**a**) and 100 nm (**b**).

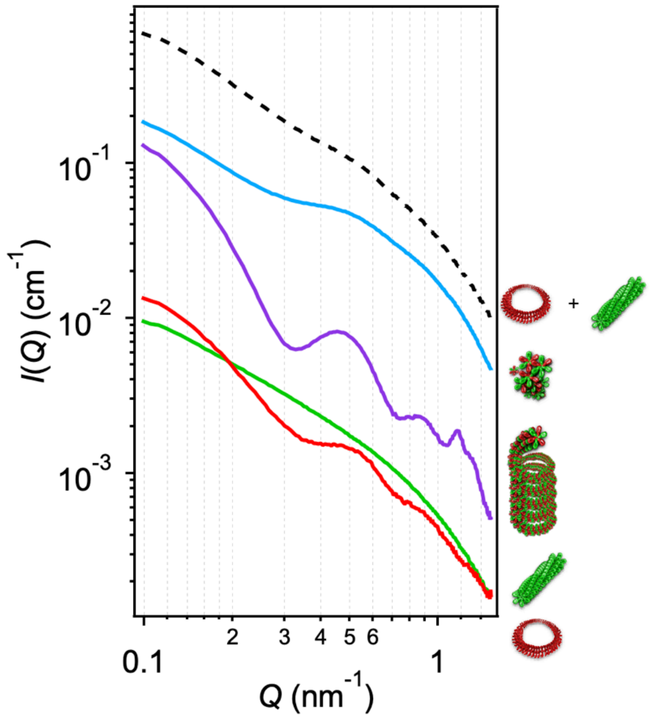

Supplementary Figure 26. SAXS profiles. SAXS profiles of **1** (250 µM; red), **2** (250 µM; green), helicoids (purple) and amorphous coaggregates (blue) of **1/2** (1:1, c_t = 500 µM). Black dotted line was obtained by summation of the individual SAXS profiles of **1** and **2**. For clarity, these profiles are offset by $I(Q)$ = 0.1 cm^{-1}.

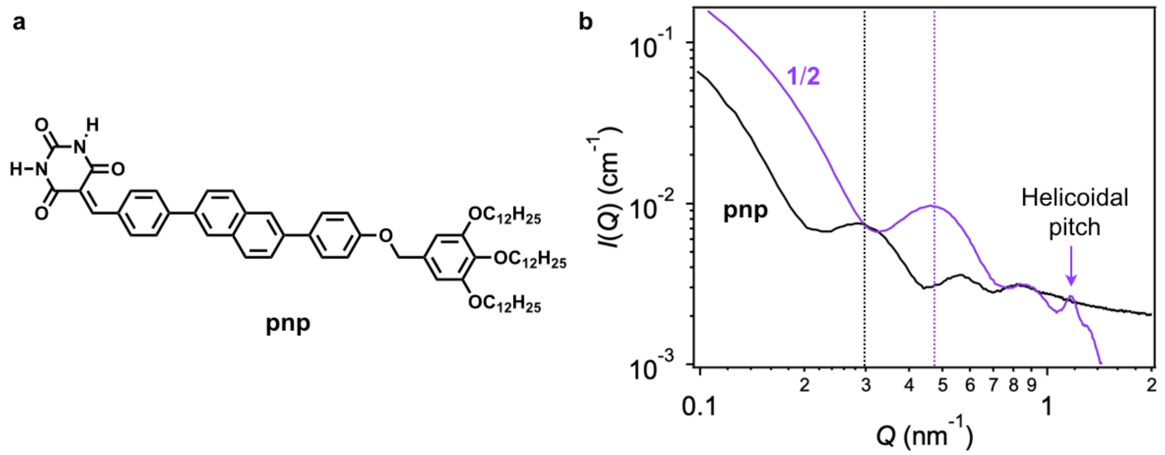

Supplementary Figure 27. Comparison of SAXS profiles of pnp and 1/2. a, Chemical structure of **pnp**. **b**, Comparison of SAXS profiles of **pnp** (black) and **1/2** (1:1; purple). Dotted lines show the positions of the first scattering peak.

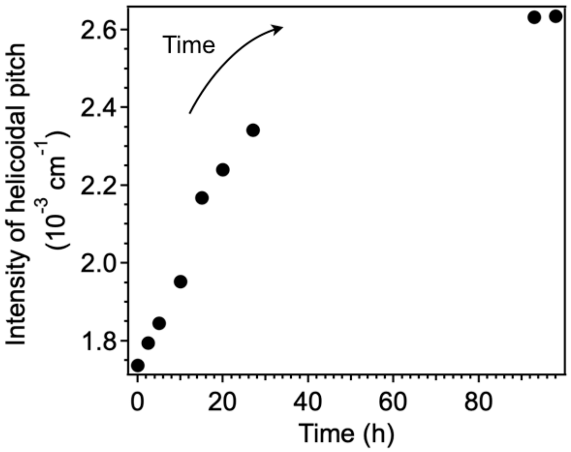

Supplementary Figure 28. Time-dependence of a SAXS peak. Time-dependent change of the intensity of the SAXS peak derived from the helicoidal pitch of **1/2** (1:1, c_t = 500 μM).

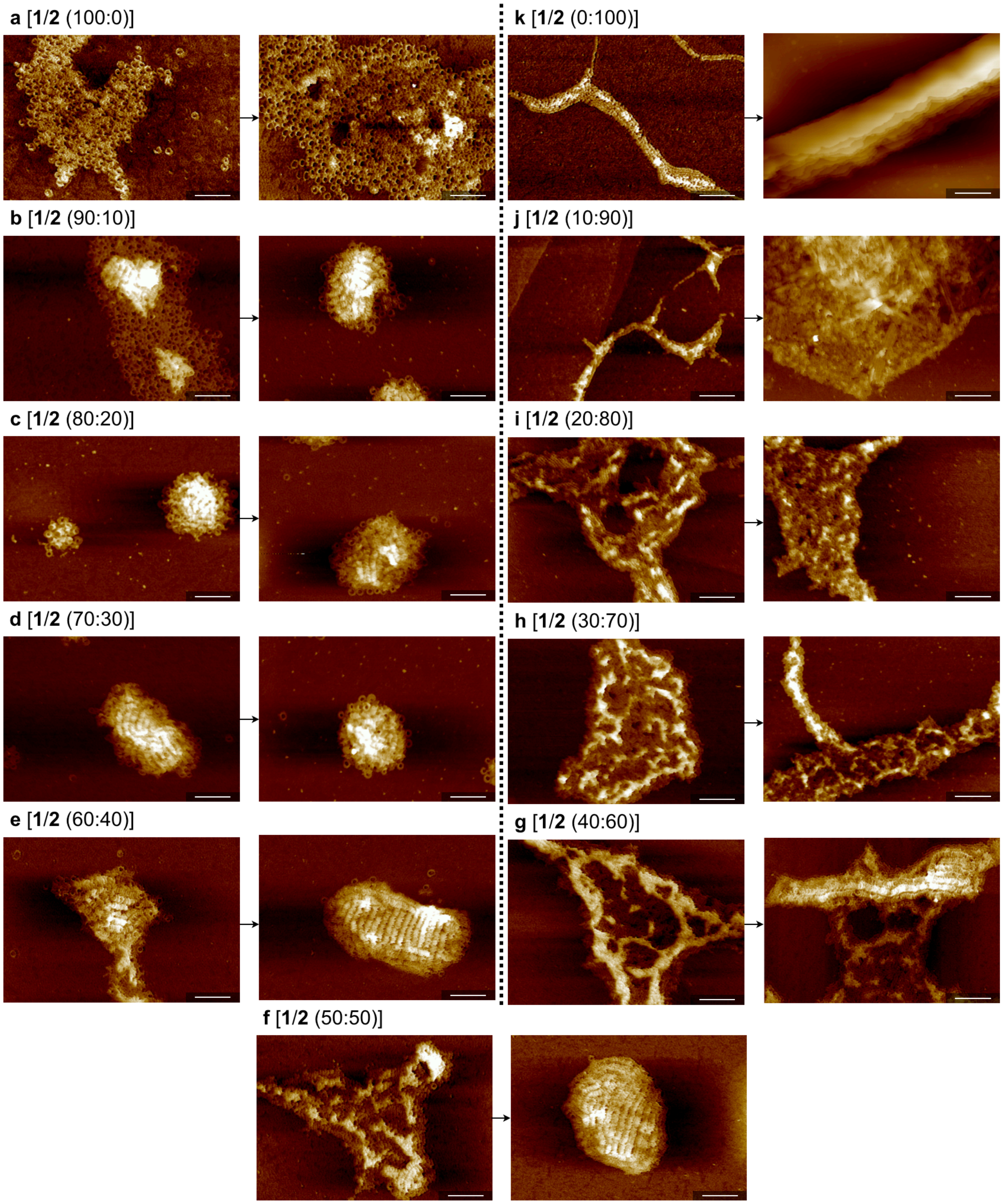

Supplementary Figure 29. AFM images of nanostructures prepared by mixing 1 and 2 with various ratios. AFM images of **1/2** with different mixing ratios while keeping c_t with 100 μM. The samples were prepared by spin-coating the solutions (10 μL) immediately after cooling (left images) and after aging for 3 days (right images). The **1/2** ratios are 100:0 (**a**), 90:10 (**b**), 80:20 (**c**), 70:30 (**d**), 60:40 (**e**), 50:50 (**f**), 40:60 (**g**), 30:70 (**h**), 20:80 (**i**), 10:90 (**j**), 0:100 (**k**). Scale bars, 100 nm. The right in (**e**) and (**g**) are the same AFM images with Fig. 5a,b, respectively.

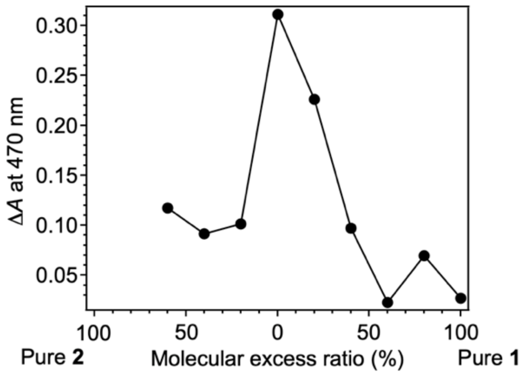

Supplementary Figure 30. Dependence of absorbance change at 470 nm on 1:2 ratio. Degree of the absorbance change ($\Delta A = A_{t=0\,\mathrm{h}} - A_{t=65\,\mathrm{h}}$) at 470 nm after 65-h aging of **1**/**2** with different mixing ratios while keeping c_t with 100 μM. The data of the pure **2** could not be recorded due to precipitation.

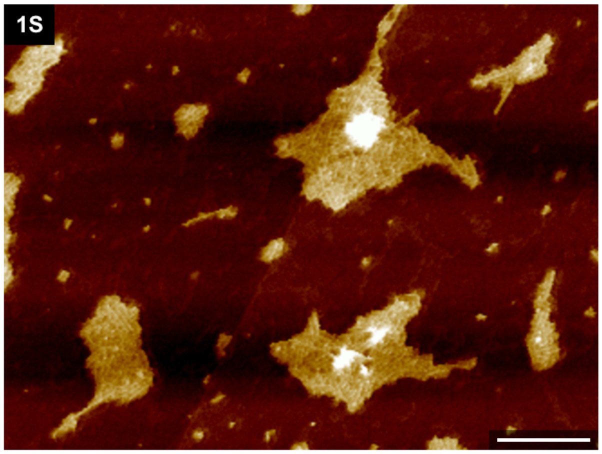

Supplementary Figure 31. AFM image of 1S. AFM image of **1S** spin-coated from a MCH solution (c_t = 250 μM) immediately after cooling. Scale bar, 100 nm.

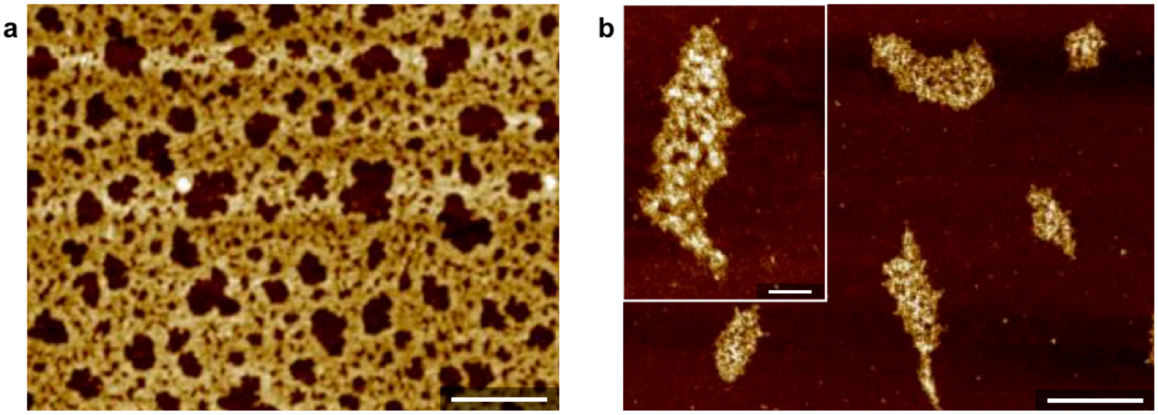

Supplementary Figure 32. AFM images of 2S. AFM images of **2S** spin-coated from MCH solutions (**a**, c_t = 500 μM; **b**, c_t = 100 μM) immediately after cooling. Scale bars, 300 nm and 50 nm (inset).

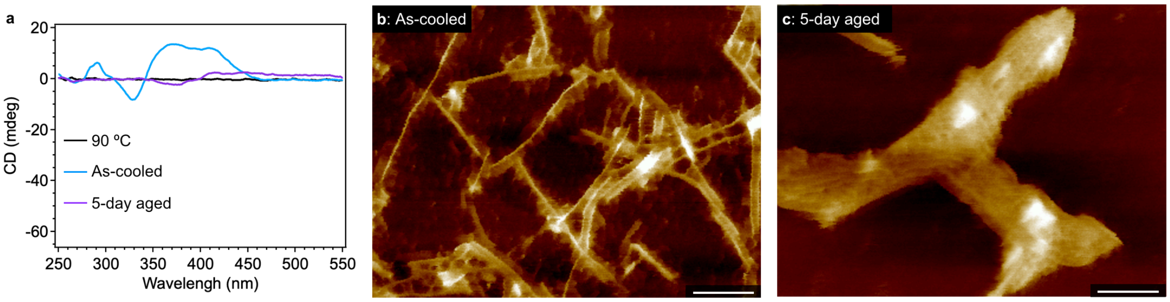

Supplementary Figure 33. CD spectra and AFM images of 1S/2. a, CD spectra of **1S/2** (1:1, c_t = 500 μM) measured at 90 °C (black), 20 °C immediately after cooling (blue), and after subsequent aging for 5 days (purple). The attenuation of the CD signal after aging is due to precipitation. **b**,**c**, AFM images of **1S/2** (1:1, c_t = 500 μM) spin-coated from the solution immediately after cooling to 20 °C (**b**) and after aging for 5 days (**c**). Scale bars, 50 nm.

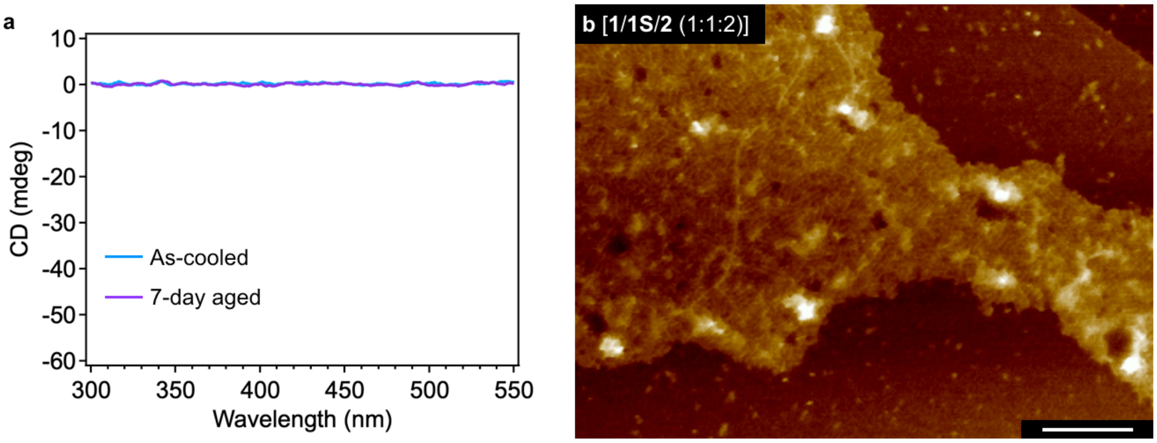

Supplementary Figure 34. CD spectra and AFM images of 1/1S/2. a, CD spectra of **1/1S/2** (1:1:2, c_t = 500 μM) measured immediately after cooling to 20 °C (blue) and after subsequent aging for 7 days (purple). **b**, AFM image **1/1S/2** (1:1:2, c_t = 500 μM) spin-coated from the solution after aging for 1 day. Scale bar, 100 nm.

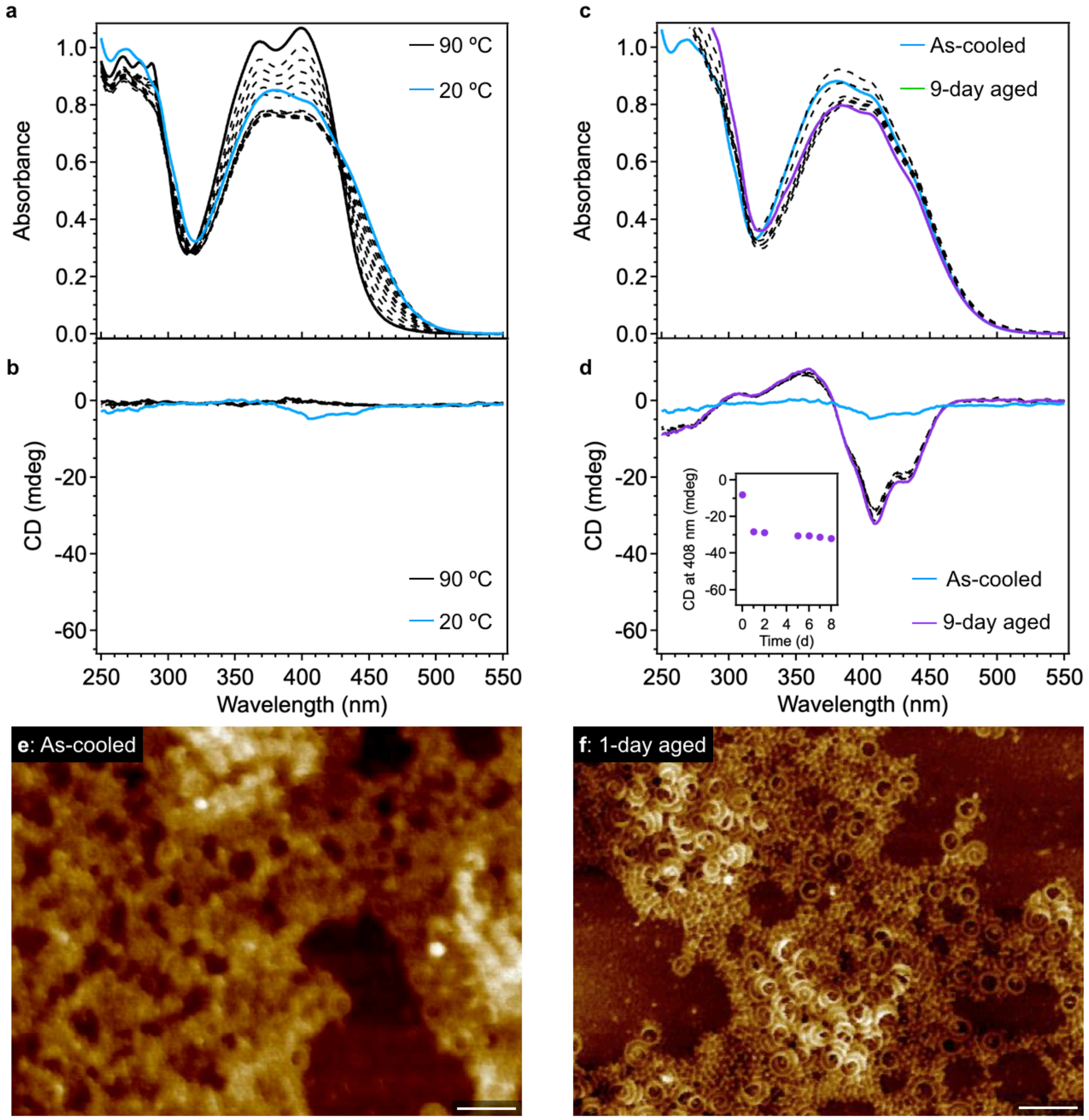

Supplementary Figure 35. UV–vis absorption, CD and AFM studies of 1/2S. a,**b**, Temperature-dependent absorption (**a**) and CD (**b**) spectra of **1/2S** (1:1, c_t = 500 μM) upon cooling from 90 °C (black) to 20 °C (blue). **c**,**d**, Time-dependent absorption (**c**) and CD (**d**) spectra of **1/2S** (1:1, c_t = 500 μM) from the as-cooled state (blue) to the 9-day aged state (purple). Inset in (**d**) shows the time-dependent change of CD intensity at 408 nm. **e**,**f**, AFM images of the above binary mixture spin-coated from the solution immediately after cooling (**e**) and after aging for 1 day (**f**). Scale bars, 100 nm.

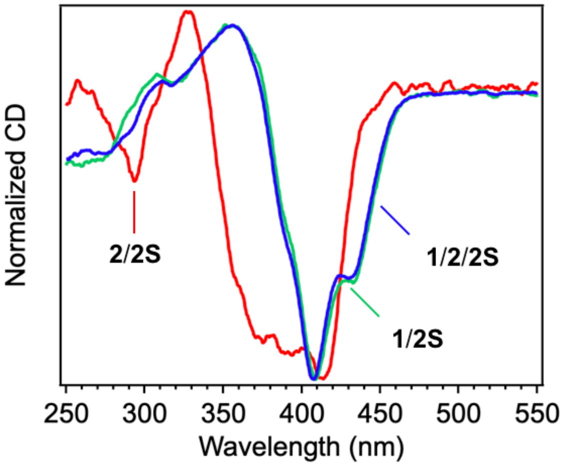

Supplementary Figure 36. Comparison of CD spectra of binary and ternary mixtures. Normalized CD spectra of **1/2S** (1:1; c_t = 500 μM; green line), **2/2S** (1:1; c_t = 250 μM; red lines) and **1/2/2S** (2:1:1; c_t = 500 μM; blue line). The spectra were recorded after equilibration.

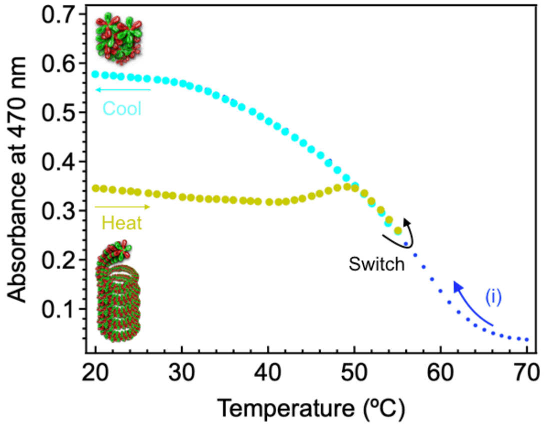

Supplementary Figure 37. Temperature-dependence of absorbance of 1/2 at 470 nm. Plots of absorbance at 470 nm as a function of temperature for **1/2** (1:1; c_t = 100 μM) in the first cooling process (blue), in the heating process after aging (yellow), and in the cooling process switched from the heating process at 55 ºC (light blue). Heating and cooling rate is 1 °C min^{-1}.

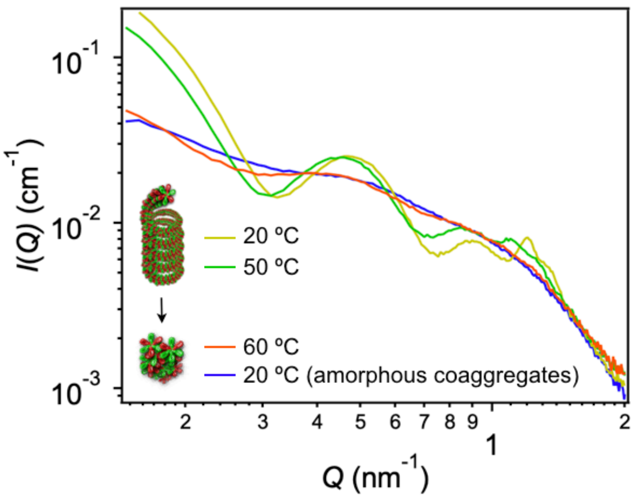

Supplementary Figure 38. Temperature-dependent SAXS profiles of helicoid. Change of SAXS profiles of the equilibrated **1/2** (1:1, c_t = 500 μM) upon heating from 20 °C (yellow line) to 50 °C (green line), and then to 60 °C (orange line), and then cooling to 20 °C (blue).

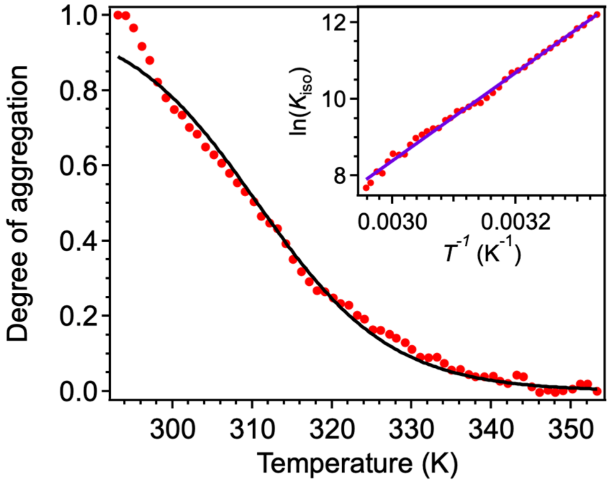

Supplementary Figure 39. Heating curve of helicoid at a low concentration. Plots of the degree of aggregation of **1/2** (1:1, c_t = 10 μM) in MCH as a function of the temperature upon heating (heating rate: 1 K min^{-1}). The solution was prepared by diluting the 7-day aged solution from 100 μM to 10 μM. The black solid line was obtained by fitting the plots to the isodesmic model, from which the melting temperature defined as the temperature for which degree of aggregation = 0.5 (T_m) and the ΔH_e that is the enthalpy release during elongation are estimated for 311 K and −106 kJ mol^{-1}, respectively. Inset shows the corresponding van't Hoff plot obtained by plotting natural logarithm of equilibrium constant (K_{iso}) as a function of T^{-1}. Purple line shows a linear fitting.

Supplementary Table

Supplementary Table 1. Thermodynamic parameters. The standard enthalpy ($\Delta H°$), entropy ($\Delta S°$), and Gibbs free energy ($\Delta G°$) of **1**, **2** and **1/2** obtained by a van't Hoff analysis in Supplementary Figs 7, 9, 28.

	$\Delta H°$ (kJ mol^{-1})	$\Delta S°$ (J mol^{-1} K^{-1})	$\Delta G°$ (kJ mol^{-1}) at 298 K
**1**	−72	−139	−31
**2**	−58	−92	−31
**1/2** (amorphous coaggregate)	−55	−88	−29
**1/2** (helicoid)	−96	−218	−32

Supplementary References

S1. Debye, P. Zerstreuung von Röntgenstrahlen. *Ann. Phy*. **351**, 809–823 (1915).
S2. Hansen, S., Calculation of small-angle scattering profiles using Monte Carlo simulation. *J. Appl. Cryst.*, **23**, 344–346 (1990).
S3. https://debyer.readthedocs.io/en/latest/
S4. Yagai, S. *et al.* Self-Organization of Hydrogen-Bonding Naphthalene Chromophores into J-type Nanorings and H-type Nanorods: Impact of Regioisomerism. *Angew. Chem. Int. Ed.* **51**, 6643–6647 (2012).
S5. Prabhu, D. D. *et al.* Self-folding of supramolecular polymers into bioinspired topology. *Sci. Adv.* **4**, eaat8466 (2018).
S6. Martin, R. B. Comparisons of Indefinite Self-Association Models. *Chem. Rev.* **96**, 3043–3064 (1996).
S7. Jonkheijm, P., van der Schoot, P., Schenning, A. P. H. J. & Meijer, E. W. Probing the solvent-assisted nucleation pathway in chemical self-assembly. *Science* **313**, 80–83 (2006).
S8. Smulders, M. M. J. *et al.* How to distinguish isodesmic from cooperative supramolecular polymerisation. *Chem. Eur. J.* **16**, 362–367 (2010).
S9. Lohr, A., Lysetska, M. & Würthner, F. Supramolecular Stereomutation in Kinetic and Thermodynamic Self-Assembly of Helical Merocyanine Dye Nanorods. *Angew. Chem. Int. Ed.* **44**, 5071–5074 (2005).
